# Three-dimensional reconstruction of renal tumor anatomy for preoperative planning of robotic partial nephrectomy in renal cell carcinoma cases with duplex kidney: a case report

**DOI:** 10.1186/s13256-024-04582-4

**Published:** 2024-05-28

**Authors:** Tuan Thanh Nguyen, Minh Sam Thai, Quy Thuan Chau, Ryan W. Dobbs, Ho Yee Tiong, Duc Minh Pham, Ho Trong Tan Truong, Kinh Luan Thai, Huynh Dang Khoa Nguyen, Thanh Thien Huynh, Huu Phuoc Le, Xuan Thai Ngo

**Affiliations:** 1 University of Medicine and Pharmacy at Ho Chi Minh City, 217 Hong Bang Street, Ward 11, District 5, Ho Chi Minh City, Viet Nam; 2 Cho Ray Hospital, Ho Chi Minh City, Viet Nam; 3 University of California Irvine, Irvine, USA; 4 Cook County Health and Hospitals System, Chicago, IL USA; 5 National University Hospital, Singapore, Singapore

**Keywords:** Duplex kidney, Artificial intelligence, Renal cell carcinoma, Robot-assisted partial nephrectomy, Robotic surgery

## Abstract

**Background:**

The duplex kidney is one of the common congenital anomalies of the kidney and urinary tract. We present two cases of renal tumor accompanied with ipsilateral duplex kidney. The image of the tumor, renal artery system and collecting system were rendered by AI software (Fujifilm’s Synapse^®^ AI Platform) to support the diagnosis and surgical planning.

**Case presentation:**

Two Vietnamese patients (a 45-year-old man and a 54-year-old woman) with incidental cT1 renal cell carcinoma (RCC) were confirmed to have ipsilateral duplex kidneys by 3D reconstruction AI technique. One patient had a Renal score 9ah tumor of left kidney while the other had a Renal score 9 × tumor of right kidney in which a preoperative CT scan failed to identify a diagnosis of duplex kidney. Using the Da Vinci platform, we successfully performed robotic partial nephrectomy without any damage to the collecting system in both cases.

**Conclusion:**

RCC with duplex kidneys is a rare condition. By utilizing a novel AI reconstruction technique with adequate information, two patients with RCC in duplex kidneys were successfully performed robotic partial nephrectomy without complication.

## Introduction

Renal cell carcinoma (RCC) is the most prevalent form of kidney cancer, accounting for 90–95% of cases, and is widespread in Western countries [1, 2]. Despite comprising only 3% of overall cancer cases, it is estimated that there are over 400,000 new diagnoses annually, resulting in nearly 180,000 fatalities attributed to renal cell carcinoma [3]. The duplex kidney is the most common congenital anomaly of the urinary tract, however it is mostly asymptomatic and rarely diagnosed in an otherwise healthy individual. However, the presence of RCC in a duplex kidney is rare and has been likely underreported in the scientific literature [4–6].

The advent and progression of advanced imaging techniques, including Computed Tomography (CT scan) and Magnetic Resonance Imaging (MRI), have significantly improved the diagnosis and surgical planning for renal tumors [2]. Often, the primary treatment option for localized renal tumors is surgical removal with partial or radical nephrectomy. In cases requiring surgical management, particularly for T1 or T2 tumors in patients with solitary kidneys or underlying chronic kidney disease, partial nephrectomy is the recommended approach when technically feasible [7, 8].

In certain scenarios, such as when dealing with anomalous kidney anatomy, comprehensive three-dimensional reconstruction and visualization of renal structures become imperative [1]. Recent years have witnessed the emergence of artificial intelligence (AI) alongside the exponential growth of medical databases and advancements in supercomputing systems. These developments have opened new horizons for AI applications in the field of medicine, including medical imaging. AI has proven to be a valuable adjunct, enhancing our capacity to extract critical insights from medical imaging data [9].

These advancements in imaging modalities and AI technologies collectively contribute to more precise diagnosis, better surgical planning, and improved patient outcomes in the realm of renal tumor management. Currently, there is a lack of published literature detailing the role of 3D reconstruction in the preoperative assessment of congenital anomalies concurrent with renal cell carcinoma (RCC). Herein, we report two cases of patients with RCC present in duplicated kidneys, aided by Fujifilm’s Synapse^®^ Artificial Intelligence (AI) Platform that helped us clearly identify the anatomical abnormalities. Leveraging this valuable information, we devised a surgical plan for partial nephrectomy with robotic assistance for both patients. This case is presented in line with the Consensus Surgical Case Report (SCARE) guidelines [10].

## Report of 2 cases

### Case 1

A 45-year-old Vietnamese male patient was incidentally discovered to have a left kidney mass during a routine health check. The patient had a history of hypertension and no other complaints. The CT scan revealed a distinct, confined mass lesion at the upper pole of the left kidney, measuring 32 × 33 × 35 mm, suggestive of RCC. The left kidney was entirely duplex on imaging. We utilized the Fujifilm’s Synapse^®^ Artificial Intelligence (AI) Platform to reconstruct a 3D image of both kidneys and clearly observed the fully duplicated left kidney along with the tumor at the upper pole. Using Tc-99 m DTPA isotope scan, the estimated split renal function of the left kidney was 52.6%, with a glomerular filtration rate of 40.3 ml/minute.

**Fig. 1:**
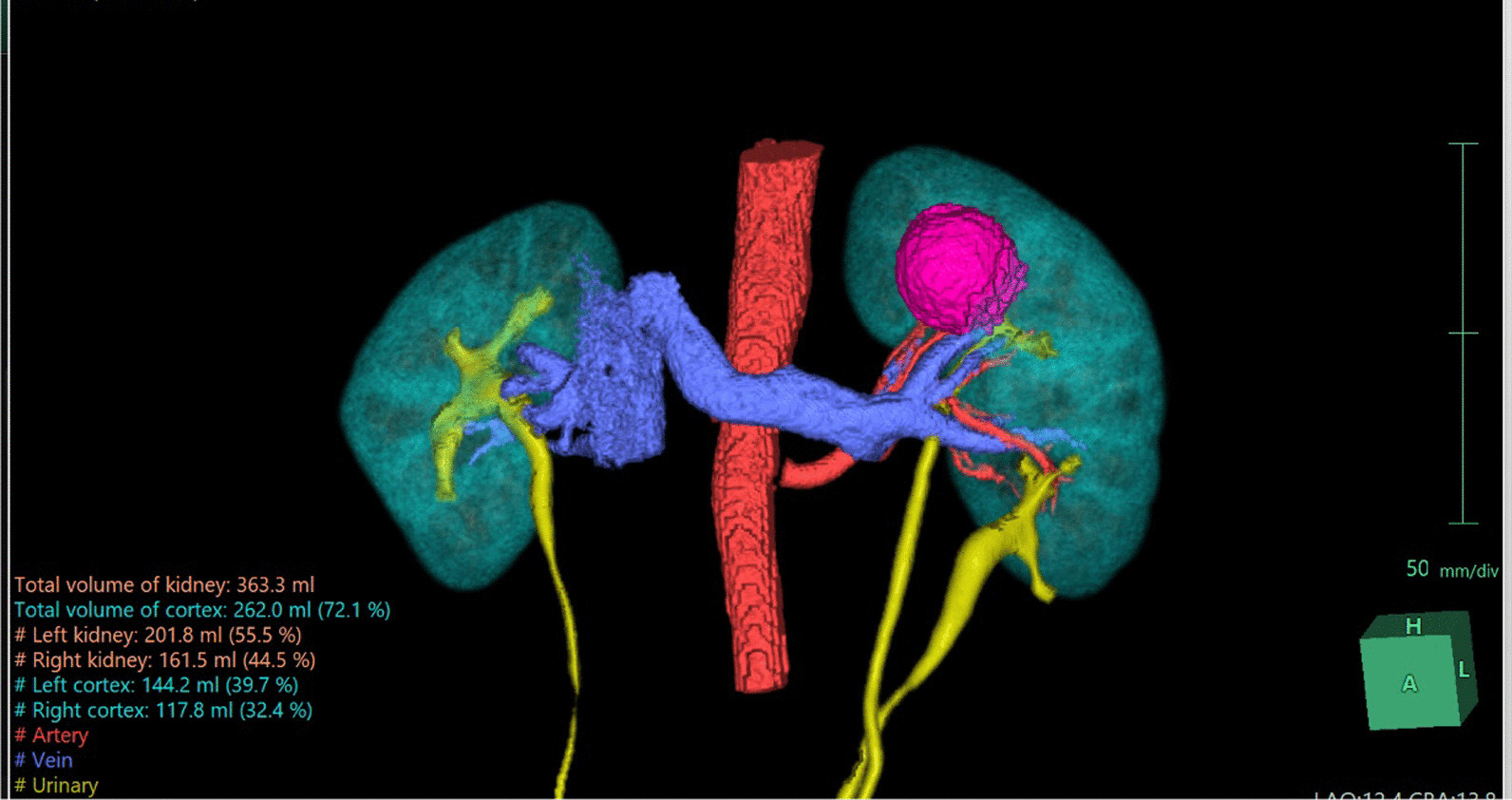
Fujifilm’s Synapse^®^ AI Platform has reconstructed the imaging system (highlighted in yellow) aiding in detecting the tumor on the entirely duplicated left kidney

The diagnosis in this case is cT1aN0M0 hilar renal tumor on the entirely duplicated left kidney, Renal score was 9ah. The patient underwent a partial nephrectomy of the left kidney with the assistance of the da Vinci robotic system. During the surgery, we exposed and observed the anatomical structure of the left duplex kidney as seen in the 3D reconstructed image (Fig. 1). The renal tumor appeared distinctly with well-defined borders and was located at the upper pole of the left kidney. We clamped the left renal artery using a Bulldog clamp and proceeded to perform an enucleation of the renal tumor. The warm ischemia time was 23 minute, estimated blood loss was approximately 50 ml, and the total surgical time was 140 minute.

**Fig. 2:**
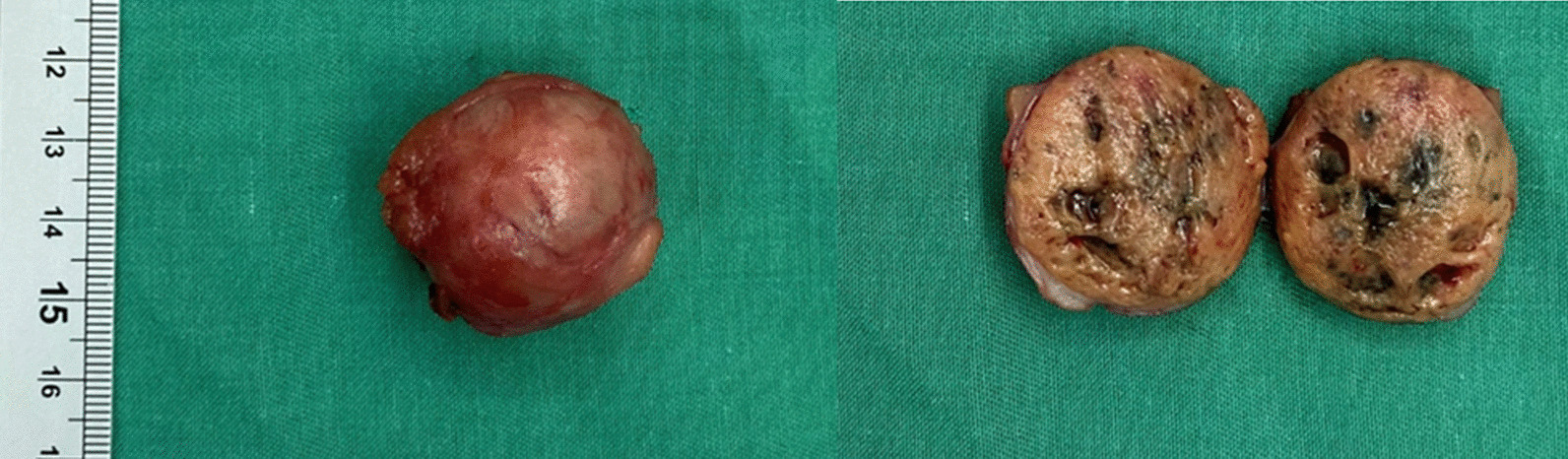
Representative image of the renal tumor after surgery

The patient was discharged after 3 days with no postoperative complications observed. The histopathological results confirmed renal cell carcinoma (RCC), with no tumor cells found at the surgical margins, indicating clear resection (Fig. 2). One-month follow-up, the renal function was preserved without change in the serum creatinine level.

### Case 2

A 54-year-old Vietnamese female patient was incidentally found to have a right kidney mass during a routine health examination. The CT scan imaging was strongly suggestive for a solid renal mass, likely renal cell carcinoma (RCC). Preoperative tests showed normal results, and both kidneys demonstrated comparable function. The left kidney had a glomerular filtration rate of 50.9 ml/minute, constituting 50.1% as determined by the Tc-99 m DTPA renal isotope study.

With the assistance of *Fujifilm’s Synapse*^*®*^* AI Platform*, we obtained detailed images of the tumor anatomy, including the blood vessels supplying the tumor. Additionally, the AI platform helped identify the presence of bilateral duplicated kidneys, a detail that was initially overlooked in the conventional CT scan (Fig. 2).

**Fig. 3:**
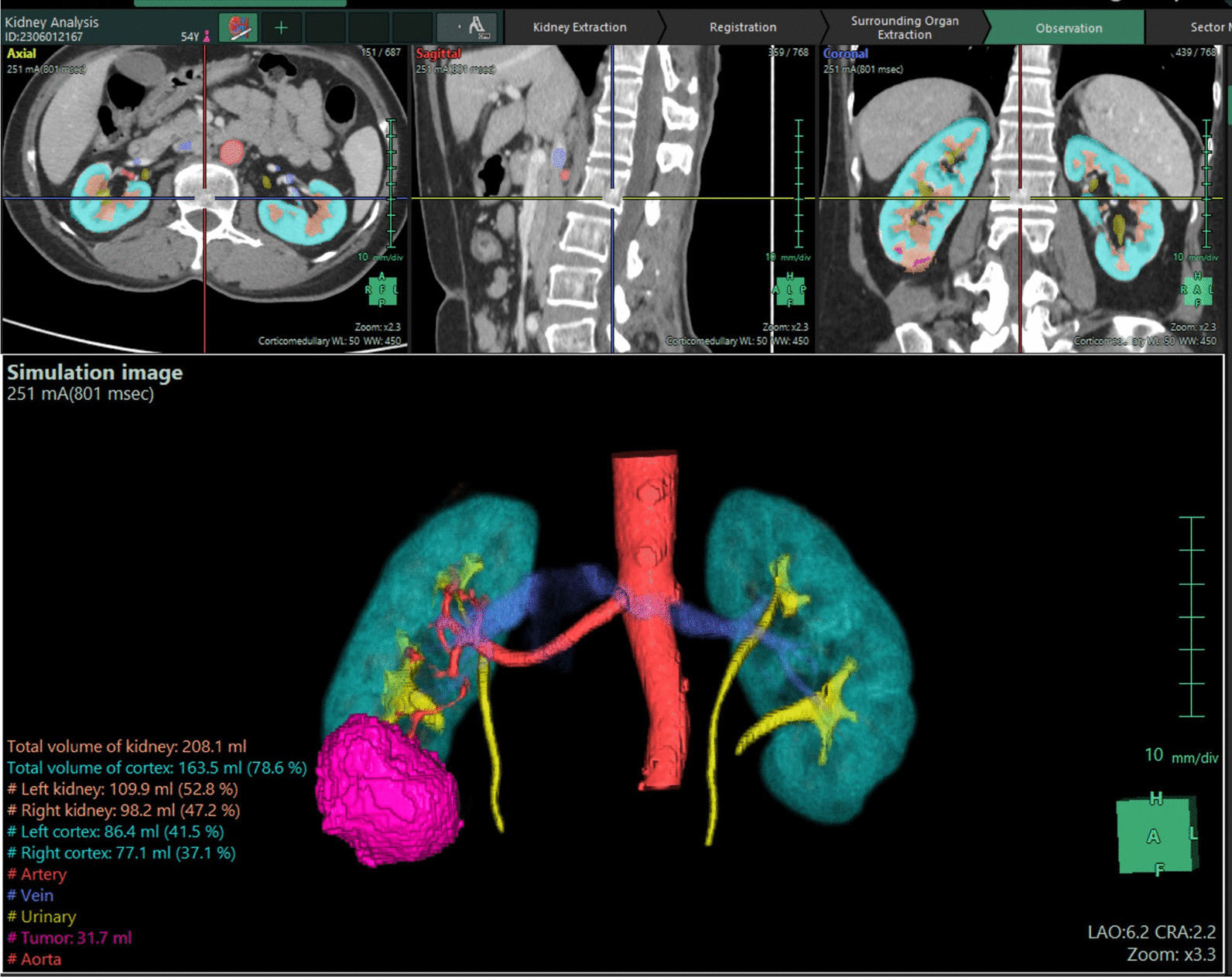
Fujifilm’s Synapse^®^ AI Platform reconstructed the imaging system (highlighted in yellow) aiding in detecting the bilateral renal tumor on the right kidney

**Fig. 4:**
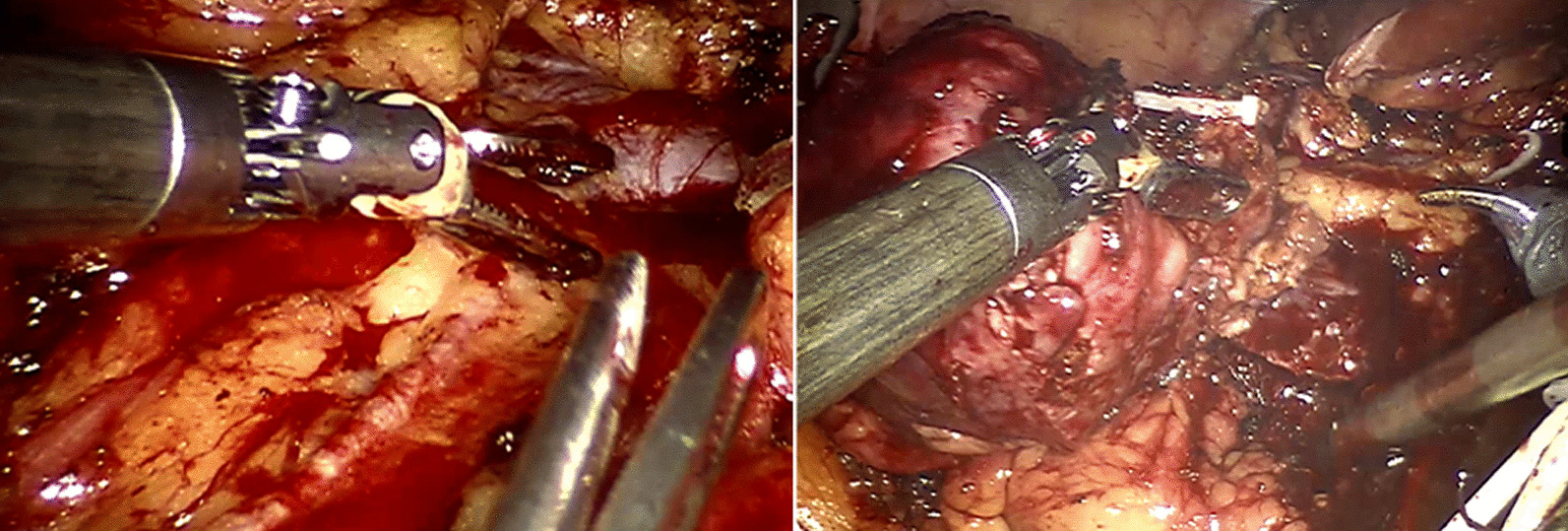
The duplicated ureters (left) and resected renal tumor (right)

The patient was diagnosed with RCC, clinically staged as cT1bN0M0, and had a RENAL score of 9x. A partial nephrectomy of the right kidney was performed using the da Vinci robotic system. During the surgery, distinct images of the two collecting systems were observed, clearly depicted in the 3D reconstructed image using AI (Figs. 3 and 4). The tumor was prominently located at the lower pole, showing well-defined borders with the normal parenchyma and no invasion into the underlying collecting systems. Examination of the renal hilum revealed a perfect match between the actual branching pattern of the blood vessels and the image generated by Fujifilm’s Synapse^®^ AI Platform.

We clamped the renal artery using a laparoscopic Bull-dog clamp and proceeded to perform an enucleation of the renal tumor. The warm ischemia time was 28 minute, estimated blood loss was approximately 60 ml, and the total surgical time was 150 minute.

**Fig. 5:**
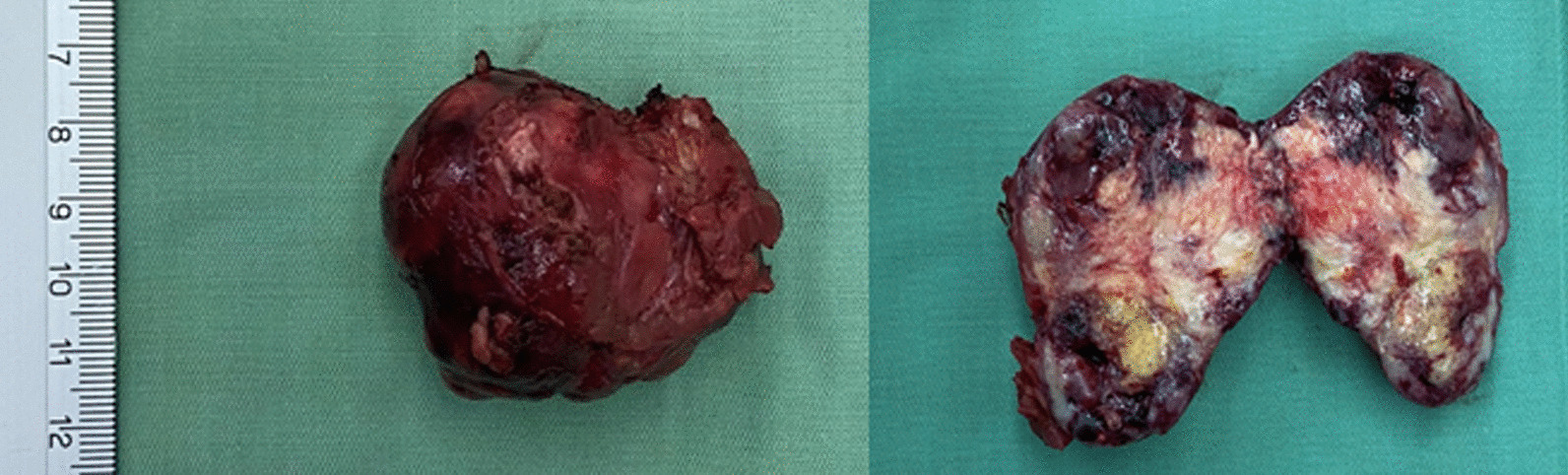
Representative image of the renal tumor after removal

The patient was discharged after 4 days with no postoperative complications observed. The histopathological results confirmed renal cell carcinoma (RCC), with no tumor cells found at the surgical margins (Fig. 5). Furthermore, the serum creatinine level was unchanged one month postoperatively.

## Discussion

Duplex kidney is the most common congenital anomaly of the urinary system, with a prevalence of approximately 0.8–1% in the general population, often detected incidentally due to the lack of symptoms [5, 6, 11]. Although RCC is the most common kidney cancer, RCC in duplex kidneys is an exceedingly rare condition [4, 6]. In adults, the first reported case of RCC in a duplicated kidney was in 2014 by Mohan *et al.,* involving a 49-year-old male patient who underwent radical right nephrectomy [4]. The first pediatric RCC in a duplex kidney was also found in a 5-year-old girl with cT3aN1M0 disease [5].

In these two cases, we have successfully integrated the latest advancements in medical technology with artificial intelligence and robotic surgery to proactively plan the surgical approach for each patient. This is the first report in the literature highlighting the application of AI in the diagnosis and treatment of RCC in duplicated kidneys with robotic assistance, aiming to preserve renal function.

In this clinical scenario, with the assistance of SYNAPSE 3D, we accurately identified the presence of tumors on the duplicated kidneys. Furthermore, the 3D simulation images helped in identifying the feeding vessels to the tumors and provided a clear visualization of the spatial relationship between the tumors and adjacent structures [1]. This information was crucial in planning the surgical approach effectively. These minimally invasive methods, in addition to ensuring equivalent oncological safety for localized tumors, offer advantages in terms of reduced invasiveness, decreased blood loss, and shorter hospitalization periods [3].

In the realm of medical imaging and preoperative planning, the use of simulation images is paramount. These images aid surgeons to comprehensively understand complex organ structures and intricate vascular systems within the human body. Traditionally, the creation of simulation images involved manual tracing and extraction from computed tomography (CT) scans. However, this method posed challenges, including operator-dependent variations and inconsistencies arising from differences in scan timing and contrast conditions [12]. Such discrepancies could compromise the accuracy of preoperative planning, a critical aspect of surgeries that are performed frequently in clinical practice [13, 14]. The SYNAPSE 3D, an advanced software solution offered by Fujifilm, excel in analyzing target and non-target areas separately and applying density and shape models to images of the target organ. The result is a significant reduction in operator-dependent discrepancies, leading to highly objective simulation images.

The kidney, often likened to a mass of intricate vessels, poses a unique set of challenges for preoperative simulation. Achieving high-quality, accurate simulation images for renal procedures is of paramount importance. These images serve as invaluable guides for surgeons, allowing them to navigate the complex vascular and parenchymal structures of the kidney with confidence and precision [15]. SYNAPSE 3D represents a significant leap forward in preoperative simulation for renal surgeries, particularly robot-assisted partial nephrectomy. This innovative platform streamlines the process of creating 3D images, significantly reducing the time required for constructing detailed vascular maps and predictive states post-tumor excision. The automation of these critical steps empowers surgeons by freeing them to manipulate and verify images during surgery itself [12]. These images derived by 3-D reconstruction may also be helpful for creating models in training simulations for educational purposes [16].

## Conclusion

Renal cell carcinoma in congenital duplicated kidneys is a rare condition, and a comprehensive evaluation of renal anatomy is essential for treatment planning. The integration of stimulation imaging AI platform into preoperative planning for robot-assisted partial nephrectomy represents a promising development. These advancements hold promise in complex clinical scenarios to enhance diagnostic precision. Further case series and cohort studies are essential to evaluate the practical applications and the cost–benefit ratio of artificial intelligence-driven 3D reconstruction platforms for standard imaging protocols in future practice.

## Data Availability

Data supporting the findings of this study are available from the corresponding author upon reasonable request.
